# Development of a Virtual Reality Platform to Promote Disaster Preparedness Among Older Adults

**DOI:** 10.1093/geroni/igab046.1360

**Published:** 2021-12-17

**Authors:** Zhen Cong, Chen Kan, Aaron Hagedorn, Sam Thomas, Reid Yeager

**Affiliations:** University of Texas at Arlington, Arlington, Texas, United States

## Abstract

This project develops a tailored and adaptive virtual reality platform to innovatively promote older adults’ disaster preparation in a socially engaging environment. The platform serves the following purposes: 1) assist older adults to develop tailored household emergency preparedness plans, 2) simulate extreme weather conditions and warnings for older adults to practice disaster response and develop relevant knowledge and skills as well as test and revise their emergency preparedness plans, 3) use the process as a social engagement tool to reduce social isolation and promote a sense of community. The virtual environments are designed in Unity to simulate extreme weather conditions/natural disasters and older adults are guided to use the HTC VR headset and experience the selected disaster scenario. The pilot VR platform will be tested among community-dwelling older adults in the Dallas-Fort Worth Metropolitan Area.

